# National

**Published:** 1896-07

**Authors:** 


*National.*—Senate Bill No. 1552, destined to retard scientific medical and surgical progress by imposing unnecessary and uncalled-for restrictions on experimental work among the lower animals, remained on the Senate calendar at the close of Congress. It will, therefore, take precedence in the next session of Congress, and it behooves all true votaries of advance along the line of progress in the medical world to use their influence with their representatives in both branches of Congress to defeat this measure.

Write your Senators and Representatives from every district.

